# Study on the construction of multi-level protection system for non-emergency transfer under the perspective of structuring

**DOI:** 10.3389/fpubh.2024.1377714

**Published:** 2025-01-15

**Authors:** Wei Liu, Ruiqiang Chen

**Affiliations:** ^1^School of Social Work, China Civil Affairs University, Beijing, China; ^2^School of Politics and Public Administration, Guangxi Minzu University, Nanning, Guangxi, China

**Keywords:** non-emergency transfer, medical security, structuring, security system, transfer services

## Abstract

**Introduction:**

The non-emergency transfer multi-level protection system is a pivotal livelihood endeavor in China, serving as a vital diversified component within the robust framework of a Chinese-style modern social security system. This system faces various challenges, including displacement of emergency capacity by non-emergency demands, uneven allocation of transfer resources, service quality variations, inadequate management structures, limited regulatory frameworks, and social acceptance issues.

**Methods:**

Leveraging structural theory, this study analyzes the primary issues in the current implementation of China’s non-emergency transfer security system. A structured approach is employed to investigate these challenges and propose solutions.

**Results:**

The study identifies key areas for improvement in the non-emergency transfer security system. It highlights the need for an enhanced internal resource allocation mechanism to boost service efficiency, skilled workforce development to improve service quality, optimized management systems and coordination mechanisms to strengthen patient confidence in recovery, and strengthened comprehensive information technology management for market oversight.

**Discussion:**

The proposed structured approach aims to foster sustainable development of the non-emergency transfer security system within a positive feedback loop. The recommendations aim to address the identified challenges and enhance the overall effectiveness of the system. By improving resource allocation, workforce skills, management systems, and information technology, the study suggests fostering deeper emotional engagement and connections, ultimately contributing to the system’s long-term success.

## 1 Introduction

Compared with pre-hospital emergency transport, non-emergency transport has been characterized by a lack of uniform standards and effective supervision, leading to issues such as indiscriminate charging and the proliferation of unqualified ambulances. These challenges have garnered widespread concern across various sectors of society. Since the adoption of the Measures for the Administration of Pre-hospital Medical Emergency Treatment (hereinafter referred to as the ‘Measures’) by the National Health Planning and Family Planning Commission’s committee meeting in 2013, China has made significant strides in exploring and refining standardized diagnosis and treatment protocols, as well as the green channel process for emergency medical triage. Notably, Article 27 of the Administrative Measures explicitly prohibits emergency centers (stations) and emergency network hospitals from utilizing ambulances for non-pre-hospital medical emergency services (1). While the divestiture of non-emergency transfer services in accordance with these regulations has temporarily alleviated the demand–supply contradiction in emergency transfer services, the organizational and management framework for non-emergency transfers remains inadequate, lacking unified standards and norms. This has resulted in confusion and a lack of coordination during the transfer process, ultimately compromising service quality and transfer efficiency. In 2020, the Opinions of the Central Committee of the Communist Party of China and the State Council on Deepening the Reform of the Medical Security System emphasized the need to foster the development of a multi-level medical security system, urging the exploration and enhancement of a fair and equitable treatment guarantee mechanism (2). This underscores the urgency and importance of addressing the challenges faced by non-emergency transport. The present study introduces a novel approach to this enduring issue by contributing to the field in several key ways. Firstly, from the perspective of structural theory, it delves deeply into the construction of a multi-level safeguard system for non-emergency transport, proposing an entirely new analytical framework and construction strategies. This contribution is significant as it addresses gaps in existing research and promotes the healthy development of non-emergency transport services. Secondly, the study offers practical applications of the theoretical framework, providing actionable insights and recommendations for stakeholders involved in non-emergency transport. Finally, the research presents policy suggestions that align with the national commitment to promoting social justice, consolidating the achievements of building a moderately affluent society, and fostering social prosperity. By focusing on collaborative governance and the reshaping of the multi-level protection mechanism for non-emergency transport, this study not only reflects a profound concern for the health and life rights of patients but also underscores the necessity of such measures in contemporary society.

## 2 Interpretation of structured non-emergency transport multi-level protection system and current research status

### 2.1 Explanation of the structural theory and the multi-level protection system for non-emergency transport

#### 2.1.1 Practical analysis of the multi-level protection system for non-emergency transport

The differences and links between the non-emergency transfer system and the emergency transfer system are the focus of the study. The non-emergency transfer system refers to the process of transferring patients who do not require emergency care from the place of hospitalization to other medical institutions or home for rehabilitation, while the emergency transfer system refers to the process of transferring patients who require emergency care from the scene of an accident or the place of onset to a medical institution for resuscitation and treatment. Although both belong to the transfer system, there are obvious differences in their objectives, targets, modes of transport and requirements. Therefore, when building a non-emergency transfer system, these differences need to be clarified, and corresponding management measures and policies need to be formulated to ensure that the transfer needs of different types of patients are effectively met. The non-emergency transfer multi-level transfer protection system is built on the basis of non-emergency transfer regulations and laws and regulations, and is led by the central level for overall planning. Meanwhile, in the process of promoting the implementation of the system, it makes comprehensive use of a variety of systematic arrangements, such as commercial medical insurance, charitable mutual aid, and social assistance, in order to solve the key problems of the market’s main bodies of supply and demand in terms of funding sources and protection treatments. The main focus of the study is on the provision of services for patients who do not require emergency treatment, with the help of a diversified and hierarchical framework that defines the responsibilities of the various protection actors in a more reasonable manner. The scientific allocation of social funds, personnel management and public infrastructure is designed to alleviate the burden of disease on patients and meet their non-emergency transport needs, thereby improving their overall quality of life.

#### 2.1.2 Structured theory in a nutshell

**Figure 1:**
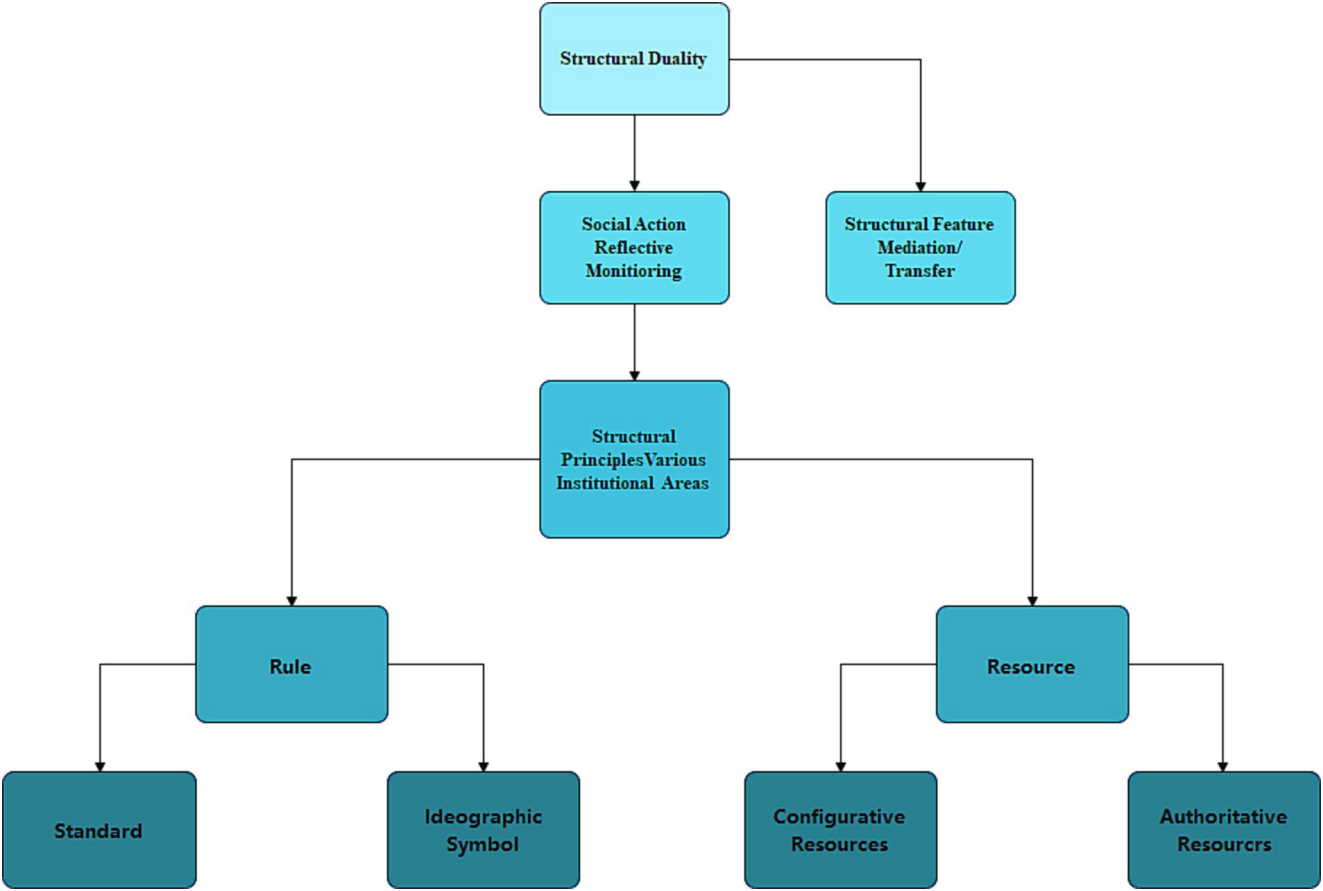
Diagram of Giddens’ theory of structural duality.

Giddens proposes structuration theory, which places action and structure in a multidimensional relational framework, aiming to dissolve the antagonistic relationship between structure and individual actors through in-depth investigation of the interaction between them. In addition, Giddens further elaborates on the concept of ‘structural duality’ in social systems (3). He argues that structures are not only shaped by human action, but also play a key role in facilitating action, i.e., they exist as mediators of action. This duality emphasizes the close relationship between structure and action, which are interdependent and affect each other. In order to reconcile these two arguments, we must explore in depth the interaction between the individual’s internal drive and the structure of the social system, as shown in Figure 1. Based on Giddens’ structuration theory perspective, structure is defined as ‘the set of rules and resources that are repeatedly involved in the process of social reproduction’. Among them, rules are understood as ‘procedural guidelines for social reproduction’, which contain two dimensions: codes of conduct and ideograms. Codes of conduct relate to norms in various fields such as politics, economics, and law, while ideograms refer to systems of symbols that convey meaning, such as language. Meanwhile, resources, as the external support conditions for social action, are divided into two categories: configurative resources and authoritative resources. Configurational resources are mainly concerned with the material resources used in the implementation of power, while authoritative resources focus on the non-material resources in the exercise of power. Giddens’ structuration theory focuses on dissecting how actions in everyday social situations are shaped by structuration and how this structural character is reproduced through the practices of actors. In the ongoing interaction between actors and structures, structures impose constraints on actors, while actors internalize these structures and exercise their subjective creativity to create new social realities within the confines of the structures. This interaction reflects the complex relationship between structures and actors, which is both restrictive and creative.

#### 2.1.3 Structured construction of a multi-level protection system for non-emergency transfers

The construction of a multi-level protection system for non-emergency transfer is a multi-dimensional and intricate social governance task, the core objective of which is to solve the notable problems faced by patients with non-emergency transfer needs in terms of transferring to hospitals for treatment, medical care and rehabilitation, and so on. This process requires the joint efforts and close cooperation of multiple participants. The establishment of the system not only covers the structural basis consisting of rule-making and resource integration, but is also deeply embedded in the strategic interactions among multiple participants. In this study, the non-emergency transport multi-level assurance system is viewed as an evolving and dynamic structure that permeates the practices of all participants and is sustained and advanced through the continuous reproduction of resources and rules. At the same time, this structure acts as a mediator, allowing the behavior of multiple actors to occur and influence each other. In addition, the structure has both a facilitating effect on the practices of social actors, i.e., it motivates them to act, and a constraining effect, i.e., it limits the scope and modalities of their behavior.

In the discussion, the government, healthcare professionals, patients, social entities, etc., are seen as diverse action entities. Through continuous self-reflective adaptation and inter-entity exchanges, these entities are continuously refining and completing the multi-level safeguard mechanism for non-emergency transport. This continuous evolution not only takes into account the allocation strategies of non-emergency transport and healthcare resources, but also profoundly influences the way in which non-emergency transport is guaranteed under the guidance of administrative rules. These factors are intertwined with each other and together they broaden the scope and level of non-emergency transfer coverage. From a traditional functionalist perspective, the multilevel system of non-emergency transfer coverage is often viewed as a “standardized” mechanism that transcends individual behaviors and is fixed and consistent. The role of comprehensive management in the construction of a multi-level protection system for non-emergency transfer cannot be overstated. Comprehensive management involves integrating various resources, such as human, financial, and technological, to ensure the effective and efficient functioning of the system. It requires coordination among different stakeholders, including government agencies, healthcare professionals, patients, and social entities. By emphasizing comprehensive management, we can ensure that the system is robust, adaptable, and responsive to the needs of patients and society at large.

However, this perspective invariably ignores the critical position of actors in actively reshaping social systems. Giddens’ structuration theory provides us with a new research perspective that reveals that the multi-level safeguarding system of non-emergency transit is constantly reshaped and reinvented in the ongoing interaction between structures and actors. As such, we should not isolate it from the practices of actors, but rather recognize the flexibility it possesses as a macro-level structured system. This flexibility may present actors with both unexpected dilemmas and opportunities for positive transformation.

Specifically: First, it is crucial to emphasize the comprehensiveness of the system design. This means encompassing multiple types of transit protection systems and a diversity of protection actors to ensure effective coverage of various needs and scenarios. In addition, maintaining a balanced and harmonious relationship between levels is central to the success of the system. This means that we should not simply add up the different levels and types of transit protection systems, but endeavor to achieve complementarity and convergence between them, while ensuring that each level has its own clearly defined function and direction of development. Finally, sustainability is crucial to the long-term development of the system. Given the heavy economic burden of patient care and the fact that the costs of mileage and the use of medical equipment during non-emergency transport have not yet been covered by medical insurance or commercial insurance, precautions need to be taken to ensure the sustainability of non-emergency transport protection. A variety of measures can be taken to achieve the goal of subjective coordination and social co-governance and to ensure the long-term stable operation of the non-emergency transport guarantee system. For example, the establishment of a special relief fund for non-emergency needs, the construction of a close network of non-emergency transport and medical cooperation, and the increase of financial subsidies. These measures are all aimed at promoting multi-party collaboration and social participation, thus ensuring the long-term and stable development of the non-emergency transport system. Through the implementation of these measures, a more comprehensive, efficient and sustainable multi-level protection system for non-emergency transport can be built to meet the needs of patients and promote the harmonious development of society.

### 2.2 Current status of research on the multi-level protection system for non-emergency transport

Research in the field of emergency medicine has been focusing on improving the standard of emergency medicine and saving medical resources at the same time, and deploying resources to form emergency units based on classification to achieve optimal efficiency (4). Unlike emergency medicine, socialized non-hospital emergency transport services are responsible for transferring patients in non-emergency situations (5). Many scholars believe that there is a huge demand for non-emergency transport services. However, the market for non-emergency transport remains chaotic and lacks effective regulation, so it is necessary to establish and regulate a non-emergency transport system (6, 7). There is a long history of non-emergency transport system construction, and different regions and countries have accumulated rich experiences and practices in this regard. In the United States, the construction of a non-emergency transport system focuses on the rational allocation and distribution of medical resources, and improves the efficiency and quality of transport through the establishment of a multi-level transport network (8). Australia focuses on the standardization and specialization of transfer services, and has improved the professional quality and service level of transfer personnel through the establishment of a unified transfer standard and training system (9).

After systematically collating the existing academic studies and practical discussions, we have identified two major shortcomings. First, from the perspective of research, previous studies have mainly focused on the assessment of the market size of the industry, and have explored more about the macro-level of non-emergency ambulance transfer services, such as the safeguard system, and the far-reaching impacts on the industry’s developmental dynamics and social behavioral patterns. However, these studies have not explored in depth how the supply and demand sides of non-emergency transfer services implement unique practices through self-examination and further adjustment in specific social contexts. As a result, there is a lack of adequate explanation and elaboration of the interaction process between the actors. Furthermore, from a disciplinary point of view, previous studies have mainly focused on the institutional level of economics or management, exploring the practical aspects of non-emergency transfer systems, while lacking in-depth thinking and analyses from a sociological point of view. In fact, the multi-level protection mechanism for non-emergency transfer of patients is constructed by government organizations and multiple social actors. By integrating various rules and resources, each actor can continuously optimize the protection mechanism in the continuous interaction. Structuration theory in the field of sociology provides new perspectives and references for solving the difficulties of transfer security on both the supply and demand sides. From this perspective, this study will explore in depth the key issues in the practice of multilevel safeguard for non-emergency transfer in China. Through in-depth analyses of the non-emergency transfer multilevel guarantee mechanism, it will reveal the interconnections among its internal elements and put forward suggestions to promote the healthy development of the system. This will help to make up for the shortcomings of existing research and provide strong theoretical support and practical guidance for the development of non-emergency transfer services.

## 3 The main problems in the practice of constructing the multi-level guarantee institutionalization of non-emergency transport

### 3.1 Reduced effectiveness of transit services due to internal misallocation of resources

The construction and promotion of a multi-level protection system for non-emergency care requires a large investment of resources of all kinds, as well as the efficient use of administrative mechanisms, social mobilization and other authoritative means, with the aim of raising the standard of protection for the transfer of patients and alleviating their financial pressures. At present, certain regions have actively used these authoritative means to make useful attempts to implement the non-emergency multi-tiered protection model, such as including some of the medical drugs required for non-emergency transfer in the insurance reimbursement scope, and enabling specific drugs to enter the basic medical insurance catalog through price negotiation. However, as a resource allocation tool, there is a limit to the carrying capacity of the health insurance fund. Within the scope of China’s medical insurance reimbursement, the types of emergency and rehabilitation medicines covered are relatively small, and most of them are classified under the Category B catalog. In addition, the supply of high-priced rehabilitation drugs in various regions still needs to be improved. In addition, the payment for rehabilitation drugs is mainly dependent on government input, with a relatively single source of funding, and the use of potential social funding sources such as commercial insurance and charitable funds is still insufficient. The government’s governance capacity in promoting the construction of a non-emergency multi-level protection system is constrained by its socio-economic development and the carrying capacity of the medical insurance fund. At the structural level, the internal imbalance between allocative and authoritative resources has led to slow progress in the construction of a non-emergency multi-tiered health insurance system. This has led to some patients who should have benefited from transfer resources are forced to forgo transfer due to financial constraints, thus weakening the overall effectiveness of non-emergency transfer protection services. For example, black ambulances lack a standardized fee schedule, and are all one-price, some seem to charge cheap fees on the surface, but once the patient is on the bus, they begin to charge additional fees of various kinds, and the stretcher has to be charged more, the oxygen and fluids have to be charged more, the nurses have to be charged more for traveling with the vehicle, and the death of the patient in the middle of the trip has to be charged more, so that the phenomenon of price increases often occurs, and families of affected patients tend to swallow their anger and find it difficult to refuse on the way.

### 3.2 Insufficient normative rules and difficult motivation of medical personnel

Normative regulations include formal systems such as laws and regulations, as well as informal systems such as habits and customs. In response to the current differences in the participation of social actors, some provinces and cities have begun to explore the construction of a multi-level non-emergency transfer protection system based on local realities. These provinces and cities have actively established special non-emergency transfer service guarantee mechanisms, thereby improving the quality of life of patients and their families. At present, a number of provinces and cities in China have successively promulgated policies and regulations on non-emergency transfer services. Specifically, non-emergency transfer policies at the provincial level have been implemented in 13 provinces and cities, such as Shanghai, Guangdong, Jiangsu, Fujian and Shandong. At the prefectural level, nine prefectures and cities (including county-level cities) in Jiangsu Province have introduced relevant management measures or opinions, while five prefectures and cities in Guangdong Province have followed suit.

However, because the State has not yet established a unified and systematic non-emergency ambulance transfer protection mechanism, there are obvious differences between regions in terms of the policy planning and actual treatment of non-emergency ambulance transfer multilevel protection systems. Such differences have led to regional imbalances in the level of non-emergency ambulance service, making it difficult to build a unified and effective multi-level protection system. In addition, non-emergency ambulance services often involve complex situations, lack of uniform charging standards and lack of accompanying medical professionals. At the same time, the incentives for medical coverage are curbed by the absence of appropriate incentive policies. In the absence of external incentives, it is difficult to evoke an intrinsic sense of responsibility among healthcare workers for the full range of treatments and patient health management, which in turn reduces the likelihood of their achieving a breakthrough in non-emergency transfer services. It is also worth noting that the existing healthcare system is largely based on the needs of common diseases, and non-emergency transport patients are often placed at the margins of this system. They usually have to undergo either long or short waiting periods to access transport services, which results in significant shortfalls in patients’ transport, treatment and rehabilitation needs. There is a general lack of independent treatment departments and corresponding healthcare delivery systems in primary healthcare that specialize in non-emergency transport services. This makes it difficult to efficiently address key issues during the transfer of non-emergency transfer patients, thus affecting the overall quality of non-emergency transfer services.

### 3.3 The articulation mechanism has yet to be perfected patient safety is lacking

First, at the national level, an organizational and management system for non-emergency transport has not yet been clearly established, which means that there is a lack of the necessary technical support to carry out the relevant services and protection work. Local governments rely heavily on their influence and decision-making capacity when promoting multi-level non-emergency transport services. At the same time, the authority for the management of transfer services is dispersed among several departments, such as the Health, Market Supervision and Administration Bureau and the Transportation Administration Bureau, and this dispersal of authority directly affects the smooth promotion of non-emergency transfer and protection work. Secondly, the mechanism of collaborative work among the various departments involved in the management of non-emergency transfer also needs to be strengthened. At present, there is a lack of effective communication and co-operation between these departments, making it difficult to form an institutional synergy to deal with the problem of non-emergency transfer. Under these circumstances, each department tends to work in its own way, making it difficult to build a unified and efficient non-emergency transfer protection system.

In addition, the mode of operation of the multi-level protection system for non-emergency transfer varies from place to place. They are mainly divided into three types: the first type is to have a portion of the capacity of the 120 emergency center divided to undertake non-emergency business, such as the 962,120 rehabilitation and discharge line opened by the Shanghai Emergency Center; the second type is to be approved by the local healthcare commission, and third-party organizations are introduced to participate in the process, such as the non-emergency transfer station in Suzhou City and the Nanjing Changke non-emergency transfer company; and the third type is more inclined to transport passenger transport, and is approved by the traffic management department Getting the qualification of net car or other related passenger transport. Nonetheless, the complementary articulation mechanism between these different tiers still needs to be improved. On the one hand, in the case of non-emergency transport services, the scope of coverage of the multiple payers involved has not yet been clearly defined; on the other hand, the functional differentiation between the different levels of the health insurance system is still unclear, which makes it difficult to give full play to its effectiveness in actual operation. In Guangzhou, for example, the multi-level protection system for non-emergency transfer services, led by commercial insurance and social assistance, fails to give full play to the function of medical assistance due to the lack of a corresponding system interface. This imperfect dual interface mechanism has a serious impact on the quality and efficiency of the transfer service system, and adversely affects the patient’s transfer experience. Patients may feel worried and anxious as a result of not receiving timely and effective treatment, which further interferes with their daily lives and mental health. This loss of “basic security” not only prevents patients from fighting their illnesses more effectively, but also poses a major challenge to the entire non-emergency transport security system.

### 3.4 Lack of monitoring of service standards and the challenge of information management

The service quality of the non-emergency transport system is directly related to the safety and health of patients. However, at present, the system lacks a unified service standard and service quality monitoring mechanism, resulting in significant differences in service quality and poor protection of patients’ rights and interests. This lack of service standardization not only harms the patient experience, but also hinders the sound development of the non-emergency transfer system. From the registration data on the network, as of the end of 2023, there were more than 5,300 enterprises engaged in non-emergency transfer services in China, of which small and micro-enterprises with less registered capital predominated, with nearly 55% of small and micro-enterprises with registered capital of less than RMB 5 million, and only 15% of those with registered capital of more than RMB 10 million. It is worth noting that until the end of 2018, the number of companies providing non-emergency transit services grew relatively slowly, increasing by only 252. However, from 2019 onwards, and especially in 2022, the number of non-emergency transfer companies grew rapidly, with more than 1,300 new companies added throughout the year, accounting for 25 per cent of the total. The increase was even more than 3,000 companies, accounting for 57 per cent of the total, during the three-year period from 2021 to 2023. By the end of 2023, Shandong, Jiangsu, Hebei, Shaanxi, Anhui and Liaoning provinces will have the largest number of non-emergency transfer companies, accounting for a combined share of more than 61 per cent, which is significantly higher than other provinces.

The non-emergency transport system involves many links and participants, and effective information management is pivotal to the seamless operation of the system. However, in practice, information management often faces problems such as scattered data, inadequate information sharing and information security risks. Patient transfer information is frequently dispersed among different healthcare organizations and transfer service providers, which makes the integration and sharing of information tricky. At the same time, as the level of informationisation continues to improve, information security issues are becoming more and more prominent, and protecting patient privacy and data security has become a serious challenge.

## 4 Non-emergency transfer multi-level protection system construction

### 4.1 Structured model of a multilevel assurance system for non-emergency transport

The establishment of a multilevel system of protection for non-emergency transport, based on a structured perspective, is both appropriate and productive. Giddens’ theory highlights that, given the inherent instability of causal mechanisms in the social sciences and the “reflexive” nature of the object of study, we need to deeply understand and effectively intervene in social phenomena through the means of “practice.” Similarly, the construction of a multi-level protection system for non-emergency transfer is a key structural reform practice in the field of public livelihood. This practice is initiated and led by the government, and at the same time calls for the participation of a wide range of actors. Structuration theory further reveals the potential of structural change and how this potential leads subjects with cognitive abilities to take appropriate actions. As China’s socialist construction moves into a new period, the structure, function and content of the non-emergency transit multi-level protection system and other elements are constantly adapting to the changes in the economic and social environment, and making corresponding changes and adjustments. The purpose of such adjustments is to better meet the welfare needs of patients at multiple levels, and to continue to attract and inspire more participants to join in the construction of the transfer protection system, and to work together to achieve the ideal state of “co-construction, co-management and sharing” in the field of health care.

**Figure 2:**
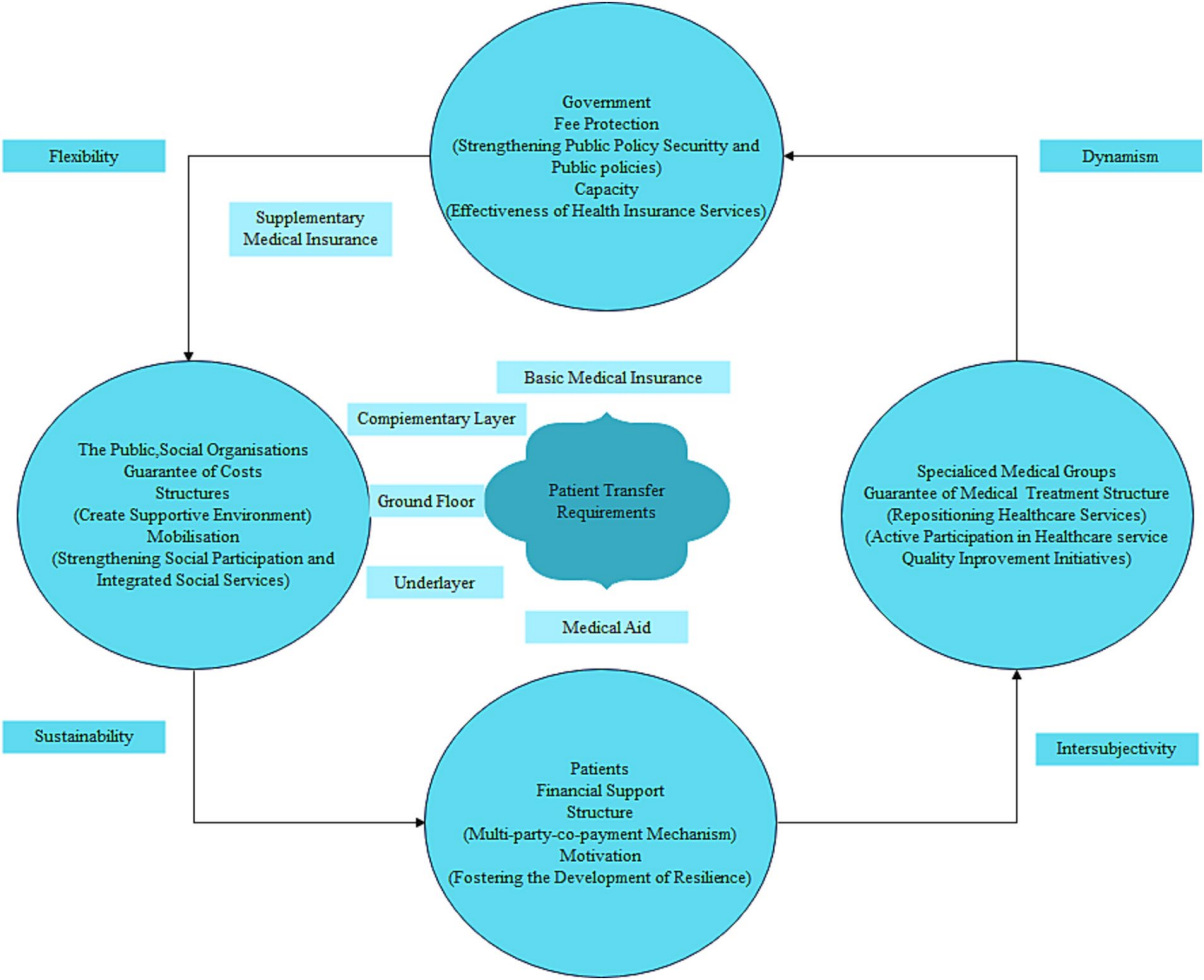
Structured view of a multi-level safeguard system for non-emergency transfers.

Aiming at the problems existing in the current practice of non-emergency transfer multilevel protection, this study starts from a structured theoretical perspective and constructs a model of non-emergency transfer multilevel protection system as shown in Figure 2. The model focuses on the transfer protection needs of patients, and through the design of the system at three levels (the main level, the complementary level, and the bottom-up level) and the synergy of the four key participants (the government, healthcare professionals, patients, the public, and social organizations), it can effectively respond to and solve the challenges and problems faced in the multilevel protection practice of non-emergency transfer. As a core component of the system, the interrelationship between structure and dynamism is always taken into account in the construction process. In addition to this, the construction of a non-emergency transfer multilevel safeguard system will help to reduce health disparities in society and increase the ability of society to take the lead in the allocation of healthcare resources, thereby effectively improving the health status and quality of life of patients. Therefore, the five core action requirements of the Ottawa Charter for Health Promotion have been incorporated into the model construction: formulating healthy public policies, creating a supportive environment, reinforcing community action, upgrading personal skills, and repositioning health services (10). At the same time, the action dimensions were appropriately adapted to take into account the dynamic complexity of specific contexts. This comprehensive approach is designed to promote the effective participation and collaboration of multiple actors in order to facilitate the continuous improvement and development of a multi-level protection system for non-emergency transport.

### 4.2 Interpretation of the relationship between the elements of the multilevel protection system for non-emergency transport

Structuration theory focuses primarily on the mutually shaping relationship between structures and actors and their dynamic evolution. In short, the various types of behavior of multiple actors are contained within a structural framework that is itself heavily influenced by behavior. Similarly, the tension between structure and action is embedded in the multilevel security system of non-emergency transport. The components of the system work in close co-operation with each other at two levels: firstly, in the internal structure of the system, it clearly defines the content and boundaries of responsibility at each level. By means of “multi-party sharing” and “collaborative social governance,” the challenges of non-emergency transport protection are jointly addressed. At the same time, this system has also given rise to the development of multiple protection bodies. For example, commercial insurance organizations and charitable mutual aid groups can find new business growth points in the process of participating in non-emergency ambulance transfer multi-level protection, thus promoting the innovation of business models. Secondly, through “reflective monitoring,” the diversified participants are able to flexibly apply the rules and resources in specific contexts, which will have a profound impact on the establishment, operation and change of the non-emergency ambulance multilevel protection system. Specifically, the non-emergency transport multilevel protection system has systematically sorted out and integrated the previously fragmented systems, and constructed a framework with a clear positioning, clear hierarchy, and synergistic work of all parts. This framework not only establishes a complementary, dynamic and stable long-term protection mechanism, but also promotes the synergistic progress of multiple protection bodies.

At the principal level, basic medical insurance plays a central role in the multi-level protection of non-emergency evacuation, giving priority to assessing and incorporating key items such as patients’ diagnosis and treatment, the use of medication and consumable technology. At the supplementary level, supplemental medical insurance, commercial health insurance, charitable donations and medical mutual aid constitute effective additions. Supplementary medical insurance includes disease diagnosis and treatment as well as specific medicines, which are included in the scope of coverage; while commercial health insurance has diversified and improved the multi-level protection system for non-emergency transport, including government-led commercial supplementary insurance for serious and serious diseases and long-term and short-term health insurance products of commercial insurance companies. Charitable donations and medical mutual aid have jointly built a mechanism for sharing and assisting in the costs of medical services and medicines for non-emergency evacuation, alleviating the problem of high prices for some innovative medicines that are beyond the economic affordability of society. In terms of protecting the lowest strata of the population, the medical assistance system provides further support for basic medical insurance, effectively safeguarding the basic rights and interests of the poor and those who have fallen into poverty as a result of illness. In addition, in the process of building this system, the government, medical professionals, patients, the public and various social organizations and other participants have formed a multilevel, nested, collaborative governance system for non-emergency transport protection. Based on their common concern for the issue of non-emergency transport protection at multiple levels, and upholding the consensus concept of “people-centered and life-first,” they continue to promote the improvement and optimisation of the multi-level protection system for non-emergency transport, and thus enhance its service level.

From a holistic point of view, the establishment of a multilevel system of protection for non-emergency ambulance transfers moves away from a rigid, static approach to management to a dynamic, adaptive system that is complex and volatile. The components of the system are interconnected and intertwined, with each participant contributing to the innovation and development of the system through routine interactive practices and “collaborative” information exchange. Thus, the multilevel assurance system for non-emergency transport is essentially a governance network that focuses on interactions between actors. Within this relatively open structure, resources and rules can circulate freely between levels, providing external assistance and internal motivation for the actions of the subjects. In turn, inter-subjective co-operation and interaction can continuously sculpt a more robust healthcare system. This process continues to evolve and is closely linked to the needs of patients and the social environment of non-emergency transport services, ultimately driving the system forward.

## 5 Recommendations for the development of a multi-level protection system for non-emergency transfers

### 5.1 Improve internal resource allocation mechanisms to enhance the efficiency of non-emergency transfer services

In a purely market-based economy, the relatively small target audience and low commercial returns of non-emergency ambulance services often make it difficult to attract funding from researchers and investment organizations, resulting in the challenge of inadequate ambulance services for patients. In order to effectively ensure the supply of non-emergency transport services, it is necessary to rely on government guidance and policy support, as well as to fully integrate and utilize the resources of various entities to meet the non-emergency transport needs of patients through the synergy of administrative structures (11).

Under the established transfer resources and operating rules, the Government should strengthen and optimize the policy of guaranteeing non-emergency transfer services, and effectively assume the leading responsibility and demonstrate its governance effectiveness. Specifically, this includes including the inclusion of medicines with clear diagnoses and efficacy in the scope of coverage of basic medical insurance, and accelerating the actual implementation of medicines that have already been included in the national medical insurance catalog, so as to ensure that patients who have already developed illnesses and received a confirmed diagnosis are able to obtain the required transport services, thereby safeguarding their rights to survival. In addition, in order to guide the rational allocation of resources for transhipment, it is necessary to establish diversified funding channels and a payment guarantee mechanism with the joint participation of many parties. For medicines and medical consumables that are expensive but have remarkable efficacy, all sectors of the community should be advocated to participate in fund-raising, and enterprises should be incentivized to provide public welfare complimentary medicines at cost price, so as to satisfy the urgent needs of special hardship groups. At present, charitable organizations are still relatively weak, and their fund-raising and mutual-aid mechanisms are not yet solid enough to provide sustained support for non-emergency transfer protection. Therefore, a new way of combining charitable assistance with medical aid should be explored to precisely help provide timely assistance to individuals incurring extraordinarily high medical expenses.

In conclusion, in the governance of non-emergency transport, the government should use “top-down” policy guidance to ensure the stable operation of the non-emergency transport system and the fair distribution of service resources. At the same time, it should also attach great importance to the actual needs and interests of patients, and consolidate the implementation of policies and improve the service effectiveness of the non-emergency transfer system through the “bottom-up” patient participation and feedback mechanism. This will not only promote the synergistic development of transfer protection and transfer services of high quality, but also more accurately meet the actual needs of patients, thereby enhancing their satisfaction and sense of gain.

### 5.2 Focus on talent team training and steadily improve service quality

Precise medical treatment and service means play a crucial role in improving the quality of life of patients and their families. Therefore, professional transporters should be committed to exploring precise transport methods and efficient medical and healthcare service systems, in order to promote non-emergency transport precision medicine and improve the quality of diagnosis and treatment services (12). At the institutional level, the state needs to formulate a comprehensive and systematic policy to protect non-emergency transport and optimize incentives for medical staff. These incentives could include financial support, research opportunities, title promotion and honors, etc., in order to stimulate the construction and growth of non-emergency transport professional teams, and to promote the continuous optimisation of patient information recording, diagnosis and treatment, tracking and monitoring, and health management. In addition, healthcare professionals should adhere to the principle of “patient-centered care,” and through a comprehensive assessment of patients’ unmet medical needs, redetermine which medical and healthcare services are more effective in improving patients’ quality of life. Currently, many countries around the world are actively building value-based healthcare systems, aiming to improve healthcare services while minimizing healthcare expenditure. It is particularly critical that healthcare providers clearly assume responsibility for the diagnosis and treatment of patients and optimize the delivery of healthcare. On the basis of assessing whether the inputs and outputs of non-emergency transfer are in balance, they will actively engage in activities to improve the quality of diagnostic and treatment services. The realization of this goal requires the establishment of close partnerships with the community, social entities and other social organizations to promote the improvement and advancement of non-emergency transfer services.

### 5.3 Optimize the management system and strengthen the articulation mechanism to boost patients’ belief in recovery

Non-emergency transport of patients is a highly comprehensive social system project. In order to ensure its efficient operation, the following three aspects are crucial: in order to promote the effective implementation of non-emergency transport safeguards, the first task is to clarify and strengthen the leading responsibilities of the people’s governments at all levels. This will be achieved through the establishment of a special organizational and management body, which will be responsible for coordinating and promoting the implementation of the various safeguard measures. At the same time, it is recommended that a leading group on non-emergency transfer protection be formed with the joint participation of multiple departments, including the Medical Protection Bureau, the Health and Wellness Commission, the Drug Administration, the Civil Affairs Bureau, the Transportation Bureau and the Market Supervision Administration. The establishment of this leading group aims to build a cross-departmental collaborative working platform to ensure that all departments can work closely together and communicate efficiently, so as to guarantee the smooth operation of non-emergency transfer work. The second point is that, given that the National Health and Health Commission has already set up a pre-hospital emergency treatment and safeguard expert committee, municipal districts should also actively try to set up comprehensive safeguard expert committees in line with local characteristics. These local committees should draw on a wide range of professionals in the fields of medicine, economics, management, health policy research, etc., so that they can pool their efforts and work together to provide patients with comprehensive and detailed protection of their health and rights. The third point is that it is also crucial to establish and improve the mechanism for the interface between the various health protection systems. This requires us to clearly define the scope of protection of basic medical insurance, supplementary medical insurance and medical assistance, so as to prevent overlapping of the contents of the protection, and at the same time ensure that they can complement and support each other. Particular attention needs to be paid to the smooth connection of non-emergency transfer services between basic medical insurance, social assistance and poverty alleviation policies, and to the careful planning of the specific directions and implementation steps for the various types of non-emergency transfer services, so as to ensure the efficient operation of the entire medical insurance system.

Through continuous improvement and optimisation of the organizational management system and the linkage mechanism, more and more patients will be able to enjoy a more comprehensive and continuous non-emergency transfer service, thus enabling their health needs to be better met. In the event of a major illness, such services can boost patients’ confidence in their recovery and enable them to cope with the challenges of illness more flexibly and efficiently. Its core philosophy is committed to protecting the health of individuals and the prosperity of communities, and the operational framework centered on resilience to adversity will help to promote the long-term benefits and sustainable development of individuals in the face of adversity and disaster.

### 5.4 Strengthen market supervision information management in all aspects and deepen emotional communication and connection

In addition to the financial pressures of treatment and medication, patients often feel a lack of emotional, informational and practical help (13). Outside of the formal healthcare system, patients prefer to obtain knowledge and emotional support from social peers and social organizations for non-emergency transport services. The public and social organizations are closely connected to patients in terms of emotional empathy and behavioral support, and together they create a supportive social environment for patients to help them alleviate the psychological stress of their illness. At the same time, these organizations actively promote health-related social culture and behavioral norms, and work to eliminate the stigma that may result from non-compliant social behavior. Based on this interactive relationship, we have constructed an interdependent social liaison mechanism to enhance patients’ social participation, pooled social resources from various parties, and provided patients with one-stop social services, including home care, education, employment and older adult care, so as to meet their needs in all aspects of life in a holistic manner. In addition, to ensure the efficiency and high quality of non-emergency transfer services, the public and social organizations need to work together to build a unified information platform for data sharing and collaboration. At the same time, it is also necessary to strengthen the training and certification of professional and technical personnel and establish a sound training system to ensure that medical and nursing personnel have professional skills and service awareness, so as to continuously improve the quality of service. In the practice of coping with various uncertainties, crises and challenges, we must constantly adjust and optimize the social support system, and commit ourselves to building a more solid and reliable social support safety network for non-emergency transfer for patients, to ensure that it covers the needs of patients in a comprehensive and seamless manner.

## 6 Conclusion

As China’s economy and social landscape continue to evolve, the non-emergency transport multilevel protection system will undoubtedly confront new opportunities and challenges. Therefore, the construction of a multi-level protection system for non-emergency transfer is a complex and multifaceted task that requires collaboration among various stakeholders. By leveraging structural theory and emphasizing comprehensive management, we can develop a system that is both effective and efficient in meeting the needs of patients and society at large. Our study has identified several key issues and challenges in the current implementation of China’s non-emergency transfer security system and has proposed a structured approach to addressing them. We believe that our recommendations will contribute to the healthy development of non-emergency transport services and ultimately enhance society’s overall well-being. Future research should continue to explore the dynamics of the multi-level protection system and how it can be further improved to meet the evolving needs of patients and society.

## Data Availability

The original contributions presented in the study are included in the article/supplementary material, further inquiries can be directed to the corresponding author.
